# SARS-CoV-2 infection in children and adolescents: a Brazilian experience

**DOI:** 10.1590/1984-0462/2022/40/2021172IN

**Published:** 2022-05-06

**Authors:** Adriana de Oliveira Ribeiro dos Santos, Bianca Rezende Lucarevschi, Mariana Hool Bajerl, Luara de Oliveira Pires, Dáfny Cristina Ubriaco, Luiz Fernando Costa Nascimento

**Affiliations:** a Universidade de Taubaté, Taubaté, SP, Brazil.; b Faculdade de Engenharia, Universidade Estadual Paulista “Júlio de Mesquita Filho”, Guaratinguetá, SP, Brazil.

**Keywords:** Coronavirus infections, COVID-19, Pandemics, Children, Adolescent, Pedriatrics, Infecções por coronavírus, COVID-19, Pandemias, Criança, Adolescente, Pediatria

## Abstract

**Objective:**

To describe clinical and epidemiological aspects of children and adolescents infected with the SARS-CoV-2 in the Municipality of Taubaté, SP, from March to November 2020.

**Methods:**

Cross-sectional study with secondary data obtained from the Epidemiological Surveillance System about confirmed cases in city residents and from medical records of patients who were treated in hospitals in Taubaté, aged between 0 and 19 years. Chi-square and Student’s t tests were used for comparisons.

**Results:**

677 cases in the studied age range were reported during the study period, corresponding to 10.1% of cases reported in the municipality. The rapid antibody test was the most used to confirm infection, followed by RT-PCR and serology. Symptoms were described in 57.7% of the cases, mainly fever and cough. Diarrhea was associated with age below 4 years, while fever, cough, headache, odynophagia, ageusia, anosmia, myalgia, and dyspnea were associated with an age ranging from 10 to 19 years. In the study period, there were no deaths from COVID-19 of residents of the municipality in the age group from 0 to 19 years.

**Conclusions:**

The study was able to identify the proportion of involvement of COVID-19 in children and adolescents in the city, and the disease had a mild evolution. The main symptoms were fever and cough, but mainly diarrhea in younger children, and headache, odynophagia, anosmia, ageusia, and myalgia in adolescents.

## RESUMO

**Objetivo:**

Descrever aspectos clínicos e epidemiológicos de crianças e adolescentes infectados pelo SARS-CoV-2 no município de Taubaté (SP) de março a novembro de 2020.

**Métodos:**

Estudo transversal com dados secundários obtidos no Setor de Vigilância Epidemiológica de casos confirmados em residentes do município e consulta de prontuários de pacientes que foram atendidos em hospitais de Taubaté, com idade entre 0 e 19 anos. Realizaram-se os testes de qui-quadrado, comparação de proporções e t de Student, sendo considerados nível de significância alfa <5%.

**Resultados:**

Notificaram-se 677 casos no período estudado, correspondendo a 10,1% do total de casos notificados no município. O teste rápido de anticorpos foi o teste mais utilizado, seguido de RT-PCR e sorologia. Sintomas foram descritos em 57,7% dos casos, sendo febre e tosse os mais frequentes. Diarreia apresentou associação com faixa etária < 4 anos, e febre, tosse, cefaleia, odinofagia, ageusia, anosmia, mialgia e dispneia tiveram associação com faixa etária de 10 a 19 anos. No período estudado, não ocorreu nenhum óbito por COVID-19 na faixa etária de 0 a 19 anos de residentes do município.

**Conclusões:**

O estudo conseguiu identificar a proporção de acometimento da COVID-19 em crianças e adolescentes no município, e a doença teve comportamento leve e boa evolução. Os principais sintomas foram febre e tosse, destacando-se diarreia nas crianças mais jovens e cefaleia, odinofagia, anosmia, ageusia e mialgia nos adolescentes.

## INTRODUCTION

On December 31, 2019, authorities of China reported to the World Health Organization (WHO) several cases of a pneumonia of unknown etiology in Wuhan, a city located in the Chinese province of Hubei. On January 30, 2020, the WHO declared that the outbreak of the disease caused by the new coronavirus (COVID-19) was a public health emergency of international importance, being characterized as a pandemic on March 11, 2020.^1^

In children and adolescents, a milder pattern of COVID-19 is observed, with few reports of severity when compared the adult population.

Although children are not considered to be at high risk of developing severe disease when infected by the new coronavirus, the sanitary measures required in the event of a pandemic have had unintended consequences for the health and well-being of children and adolescents. Closed schools, social distancing and reduction of health services supply were necessary to try and contain the spread of the disease.^2^

Regarding the pathophysiology, the expression of the primary target receptor for SARS-CoV-2, the angiotensin-2 converting enzyme (ACE-2), decreases with age.^3^ This enzyme has a protective effect on the lungs by limiting the inflammation and pulmonary capillary leakage mediated by angiotensin-2. The disease, in its severe form, is associated with high viral loads in adults. Children have a strong innate immune response due to trained immunity (secondary to live vaccines and frequent viral infections), likely leading to early infection control.^3^

Thus, epidemiological studies can help to better understand the behavior of the disease in this population and guide public health and education policies. 

The objectives of this study were to describe the clinical and epidemiological aspects of children and adolescents infected with SARS-CoV-2 in the city of Taubaté (SP) from March to November 2020.

## METHOD

**Figure 1:**
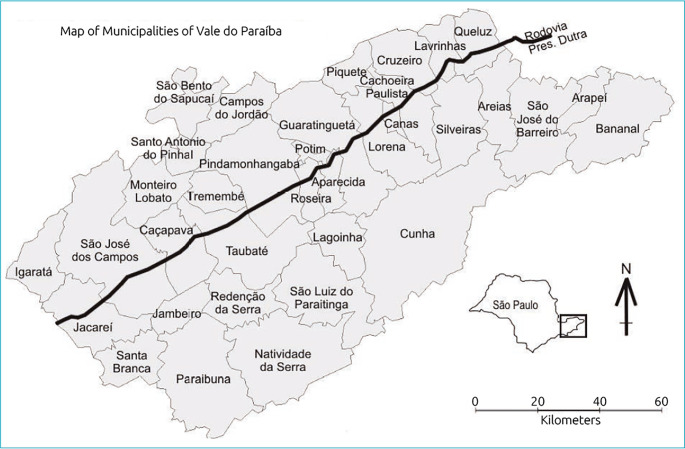
Location map of the city of Taubate^5^.

Taubaté is a municipality located in the east side of the state of São Paulo, Brazil, in a region known as Vale do Paraíba, at coordinates 23°01’S and 45°33’W, occupying an area equal to 624.9 km² with about 350 thousand inhabitants and human development index (HDI) of 0.800. The urban area occupies 91.0 km² and is crossed by the Presidente Dutra highway, an important traffic route, and by the railroad, being recognized as an industrial and technological center.^4^ The exact location of Taubaté is shown in Figure 1.^5^

A cross-sectional study was carried out based on secondary data obtained from the Sector of Epidemiological Surveillance of the Department of Health of Taubaté of confirmed cases of infection by SARS-CoV-2 in residents of Taubaté of both genders and aged between 0 and 19 years; then, the medical records of patients treated at two hospitals of Taubaté—a public and a public-private hospital—were consulted. According to the Ministry of Health, all laboratories in public or private networks must officialy notify the results of diagnostic tests to detect COVID-19.^6^ According to the recommendations of the Ministry of Health’s Epidemiological Surveillance Guide, all suspected cases of Influenza syndrome (IS), hospitalized severe acute respiratory syndrome (SARS), deaths from SARS, as well as asymptomatic individuals with laboratory confirmation of COVID-19 infection by Immunological and molecular methods.^6^

Therefore, all IS notification forms, including asymptomatic ones, and that presented any of the following positive tests for SARS-CoV-2 were considered confirmed and included in the present work: molecular biology by the RT-PCR method; immunological test by enzyme-linked immunosorbent assay (ELISA); immunochromatography (rapid test) to detect antibodies; electrochemiluminescence immunoassay (ECLIA) or antigen search by immunochromatographic method to detect antigen.

According to the Information Technology Department of the Unified Health System (DATASUS), in 2012 (the most recent information available), the municipality of Taubaté had 355,410 inhabitants, of which 83,419 were between 0 and 19 years old.^7^

The following information was extracted from the IS notification forms: biological sex, presence of fever, cough, dyspnea, anosmia, ageusia, headache, runny nose or coryza, odynophagia, diarrhea, other symptoms, comorbidities, presence of a contact and confirmation test performed.

For possible comparisons, ages were grouped as the following: 0 to 4 years, 5 to 9 years and 10 to 19 years. Tables with this information were built with respective percentages and according to age group. The distributions of age groups according to biological sex were estimated with respective proportions.

When necessary, chi-square tests, comparison of proportions and Student’s t test were performed, with significance level of alpha <0.05. 

This project was approved by the Research Ethics Committee of Universidade de Taubaté (UNITAU) under CAAE - 39637020.4.0000.5501.

## RESULTS

Between March and November 2020, 677 cases of children and adolescents aged between 0 and 19 years infected with SARS-CoV-2 were reported in the city. The total number of cases of COVID-19 in the municipality of Taubaté until November 30, 2020 was 6,709.^8^ Therefore, the studied group represents 10.1% of the total cases reported in the study period and 0.8% of the population in this age group. Age ranged from 2 months to 19 years, with a mean of 10.8 years for both biological sexes. There was a slight predominance of males (n=351, 51.8%), but without statistical significance, in the three age groups considered; 64% (n=80) of the youngest age group (0-4 years) were up to 2 years old, 41.3% (n=33) were aged 13-24 months, 47.5% (n=38) 7-12 months and 11.3% (n=9) were under 7 months of age. Table 1 shows the distributions by age, bilogical sex, signs and symptoms, comorbidities, and presence of a contact.

**Table 1:** Distribution of 677 cases of children and adolescents up to 19 years of age infected with SARS-CoV-2 in the municipality of Taubaté (SP), from March to November 2020, according to age, biological sex, signs/symptoms, comorbidities and contacts.

Characteristics	0–4 years	5–9 years	10–19 years	Total
	n=142 n (%)	n=146 n (%)	n=389 n (%)	n=677 n (%)
Male	68 (47.9)	83 (56.8)	200 (51.4)	351 (51.8)
Female	74 (52.1)	63 (43.2)	189 (48.6)	326 (48.2)
Fever	46 (32.4)	27 (18.5)	90 (23.1)	163 (24.1)
Cough	26 (18.3)	13 (8.9)	118 (30.3)	157 (23.2)
Dyspnea	3 (2.1)	5 (3.4)	38 (9.8)	46 (6.8)
Anosmia	–	5 (3.4)	58 (14.9)	63 (9.3)
Ageusia	–	4 (2.7)	65 (16.7)	69 (10.2)
Headache	3 (2.1)	14 (9.6)	110 (28.3)	127 (18.8)
Coryza	18 (12.7)	10 (6.8)	53 (13.6)	81 (12.0)
Odynophagy	5 (3.5)	16 (11.0)	108 (27.8)	129 (19.1)
Diarrhea	18 (12.7)	4 (2.7)	16 (4.1)	38 (5.6)
Myalgia	2 (1.4)	3 (2.1)	46 (11.8)	51 (7.5)
Respiratory diseases	2 (1.4)	8 (5.5)	19 (4.9)	29 (4.3)
Heart diseases	–	1 (0.7)	3 (0.8)	4 (0.6)
Other comorbidities	1 (0.7)	3 (2.1)	6 (1.5)	10 (1.5)
Unspecified contact	33 (23.2)	26 (17.8)	63 (16.2)	122 (18.0)
Family contact	41 (28.9)	71 (48.6)	128 (32.9)	240 (35.5)

The rapid antibody test was the most common test used to confirm diagnosis, accounting for 63.5% (n=430) of cases, followed by RT-PCR in 32.1% (n=217) and serology in 4.4% (n=30).

The signs and symptoms found and the p-value are described in Table 2. In 42.3% of the forms, the symptoms were not described and the patients were considered asymptomatic. Of these, laboratory confirmation was performed by RT-PCR test in 10.5% (n=30), serology in 4.2% (n=12) and rapid antibody test in 85.3% (n=244). Comorbidities were reported in 43 cases (6.3%), respiratory disorders being the most common (n=29), especially asthma (n=17), with no statistical significance between presence of comorbidities and frequency of infection.

**Table 2:** Signs and symptoms of children and adolescents up to 19 years of age infected with SARS-CoV-2 in the city of Taubaté (SP), from March to November 2020.

Characteristics	0-4 years n=142 (%)	5-9 years n=146 (%)	10-19 years n=389 (%)	Total n=677 (%)	p-value
Fever	46 (32.4)	27 (18.5)	90 (23.1)	163 (24.1)	0.024
Cough	26 (18.3)	13 (8.9)	118 (30.3)	157 (23.2)	<0.001
Dyspnea	3 (2.1)	5 (3.4)	38 (9.8)	46 (6.8)	0.021
Anosmia	–	5 (3.4)	58 (14.9)	63 (9.3)	0.006
Ageusia	–	4 (2.7)	65 (16.7)	69 (10.2)	<0.001
Headache	3 (2.1)	14 (9.6)	110 (28.3)	127 (18.8)	<0.001
Coryza	18 (12.7)	10 (6.8)	53 (13.6)	81 (12.0)	0.086
Odynophagy	5 (3.5)	16 (11.0)	108 (27.8)	129 (19.1)	<0.001
Diarrhea	18 (12.7)	4 (2.7)	16 (4.1)	38 (5.6)	<0.001
Myalgia	2 (1.4)	3 (2.1)	46 (11.8)	51 (7.5)	0.033

The medical records of 42 children and adolescents aged up to 19 years who sought medical care were analyzed, all confirmed by means of RT-PCR test; 23.3% (n=10) of cases were in children aged 0 to 4 years, 4.7% (n= 2) aged 5 to 9 years and 72% (n=30) aged 10 to 19 years. Most cases were of females (n=26, 60.5%). The most common symptom was cough (n=27, 62.8%), followed by fever (n=24, 55.8%), runny nose or coryza (n=21, 48.8%), headache (n=16, 37.2%), odynophagia (n=13, 30.2%), diarrhea (n=9, 20.9%), ageusia and anosmia (n=8, 16.3%) and dyspnea (n=4.9.3%). Comorbidities were not described in this group. None of the patients required hospitalization and/or ventilatory support.

During the study period, there were no deaths from COVID-19 in the age group 0-19 years among residents of the municipality. There have been no reports of Multisystem inflammatory syndrome in children (MIS-C) associated with COVID-19 either.

## DISCUSSION

As far as we are aware of, this was the first study carried out in the state of São Paulo and in the Vale do Paraíba region to estimate the prevalence and profile of children and adolescents infected with the new coronavirus. Most studies in this age group have been on children who needed hospitalization, who had extrapulmonary manifestations, and who evolved more severely.^9,10,11^

The first published studies demonstrated that the population under 19 years of age was 1.7% of confirmed cases of COVID-19 in the United States (USA), 2.1% in China and 6.2% in the Republic of Korea, all confirmed by RT-PCR.^12,13,14^ These numbers are lower than those found in our study, as this population represented 10.1% of all cases, being 5.5% up to 12 years of age. Data from the state of São Paulo show that the age group up to 19 years accounts for 7.5% of the total number of cases reported and 0.2% of mortality from COVID-19.^8^

There was a large proportion of asymptomatic patients in this study, and possibly they had had contact with confirmed cases and underwent examinations probably on medical advice, by active or spontaneous search. It is likely that confirmed cases are still undereported, given the milder nature of COVID-19 in children. A pilot study in the United Kingdom did not demonstrate disparity in community cases of SARS-CoV-2 infection between adults and children (0.24% of the total population and 0.30% under 19 years of age).^15^

In this study, the rapid antibody test was the most used for laboratory confirmation, which is a possible limitation of the study. In the Ministry of Health’s Epidemiological Surveillance Guide^6^, the immunochromatographic assay for rapid detection (rapid test) and qualitative IgG/IgM antibodies of the new coronavirus infection were recommended as a tool to aid in the diagnosis of the disease caused by SARS-CoV2. These are qualitative tests aimed for screening and diagnostic assistance. Negative results do not exclude SARS-CoV2 infection, and positive results cannot be used as absolute evidence of SARS-CoV2 and should be interpreted along with clinical data. Many companies produce rapid tests to diagnose COVID-19, and the product must be registered with the National Health Surveillance Agency (Anvisa) and validated by the National Institute of Quality Control in Health of the Oswaldo Cruz Foundation (INCQS/Fiocruz). Therefore, these tests are not adopted for clinical decision-making, but only for surveillance operation through population survey studies or as a diagnostic aid. Even though They are validated, rapid tests have limitations; for example, they need to be performed from the 8th day of the onset of symptoms.^6^

A meta-analysis conducted to compare the diagnostic performance of serological tests reported varying sensitivity and specificity, with better accuracy after two weeks of symptoms, concluding that these tests play an important role in supplementing the diagnosis of COVID-19, despite documented cross-reactions with other coronaviruses and the heterogeneity of different tests available.^16^ Oligosymptomatic patients may have low concentrations of antibodies, and false-negative results are more frequent.^17^

No statistical significance was found in relation to biological sex, which means it affects both sexes equally, as reported in other studies.^12,18,19,20^

Observing the distribution by age group, cases were predominant between 10 and 19 years old; among these, the symptoms were more specific and similar to those presented by adults affected by COVID-19, including fever, cough, dyspnea, odynophagia, headache, anosmia, ageusia and myalgia, as also found by other authors.^9,21^ Fever and cough are the most common symptoms reported in publications addressing children and adolescents.^22^

In younger children, up to 4 years of age, fever and cough were also the main symptoms, but diarrhea was highlighted as the most significant isolate symptom. Rabha et al. also found a high frequency of diarrhea in children under 3 years of age.^9^

The first epidemiological study considering pediatric patients with COVID-19 was carried out in China and included 731 children with laboratory-confirmed disease between January and early February 2020. The proportion of children classified as severe or critical cases was, respectively, 2.5% and 0.6%. Infants and preschool-age children were more likely to have severe clinical manifestations than older children, which is in line with previous data from children with non-SARS-CoV-2 coronavirus infections.^18^

In our study, the number of children and adolescents infected with comorbidities was relatively low, and respiratory diseases, especially asthma, were the most common comorbidities found. No comorbidities were described among patients who sought hospital medical care. Chronic lung diseases (including asthma) have been frequently found in infected patients, but it is not associated with severity.^9,12,23^ Sena et al. found a significance between lethality and the presence of comorbidities, mainly neoplasms.^20^ Lopes et al. found chronic neurological diseases as the most frequent comorbidity in patients who progressed to death.^10^

In Wuhan, of the patients who were hospitalized, 0.9% were under 15 years of age, and no deaths were reported.^24^ In the US and in the UK, children accounted for 1% and 0.9% of hospitalizations, respectively^19,21^. The first major report published by the China Centers for Disease Control and Prevention included 44,672 confirmed cases of COVID-19 and one death of a child under 19.^13^ In Italy, 1.1% of initial reported deaths occurred in people under 50 years and none of them were children.^22^

An important multicenter study including 582 patients aged up to 18 years treated in hospitals in 21 European countries, all confirmed by RT-PCR, found comorbidities in 25% of patients and the need of 8% of them for assistance in the Intensive Care Unit (ICU). Admitting young male patients with comorbidities in the ICU was also taken as a risk factor. The lethality was of 0.69%, and the authors emphasize that most participating units were part of tertiary or quaternary health institutions and that, probably, the study population mainly represents individuals more likely to present severe manifestations of the disease.^25^

What is known about the pathophysiology of the disease and the innate immune response of children and the excellent regeneration capacity of the pediatric alveolar epithelium may be a contributing factor to the milder and more favorable clinical expression of COVID-19.^3^ Children and adolescents present comorbidities less frequently. However, infants and children with pre-existing illnesses can be high-risk groups and need careful monitoring.

Joint efforts have been required to maintain the continuity of services such as health care, schools and day care centers. The response to the pandemic forced the development of strategic relationships, policy reforms and new practices. Children’s health and social systems will undergo a fundamental transformation as a result of this pandemic.^3^

Some limitations of the study were the use of secondary data sources, which may be incomplete, and the type of test used, which, in most cases, was the rapid antibody test, rulling out the possibility of confirming the time frame between the onset of symptoms and the test.

Despite limitations, our study was able to identify the proportion of involvement of COVID-19 in children and adolescents in the city, and the disease had a mild behavior and good evolution. The main symptoms were fever and cough, with diarrhea in younger children, and headache, odynophagia, anosmia, ageusia and myalgia in adolescents.

We emphasize the importance of carrying out diagnostic tests in suspected cases and of isolation measures for suspected cases and contacts, to avoid transmission (especially in more vulnerable groups), and the need to monitor the evolution of infected patients, particularly younger children with comorbidities, to detect conditions of potential greater severity.

A better understanding of the evolution of these cases is essential to improve the care provided to children and adolescents with COVID-19. At the time of completion of this study, immunization against the new coronavirus was in progress across the country and there was still no definition by the National Immunization Plan of vaccination coverage for children under 18 years of age.
